# Using prediction polling to harness collective intelligence for disease forecasting

**DOI:** 10.1186/s12889-021-12083-y

**Published:** 2021-11-20

**Authors:** Tara Kirk Sell, Kelsey Lane Warmbrod, Crystal Watson, Marc Trotochaud, Elena Martin, Sanjana J. Ravi, Maurice Balick, Emile Servan-Schreiber

**Affiliations:** 1 Johns Hopkins Center for Health Security, Baltimore, USA; 2 Department of Environmental Health and Engineering, Johns Hopkins Bloomberg School of Public Health, Baltimore, USA; 3 Hypermind, llc, New York, USA; 4 School of Collective Intelligence, Mohammed VI Polytechnic University, Ben Guerir, Morocco

**Keywords:** Forecasting, Epidemic prediction, Crowd-sourced, Infectious disease, COVID-19, Ebola, Influenza

## Abstract

**Background:**

The global spread of COVID-19 has shown that reliable forecasting of public health related outcomes is important but lacking.

**Methods:**

We report the results of the first large-scale, long-term experiment in crowd-forecasting of infectious-disease outbreaks, where a total of 562 volunteer participants competed over 15 months to make forecasts on 61 questions with a total of 217 possible answers regarding 19 diseases.

**Results:**

Consistent with the “wisdom of crowds” phenomenon, we found that crowd forecasts aggregated using best-practice adaptive algorithms are well-calibrated, accurate, timely, and outperform all individual forecasters.

**Conclusions:**

Crowd forecasting efforts in public health may be a useful addition to traditional disease surveillance, modeling, and other approaches to evidence-based decision making for infectious disease outbreaks.

**Supplementary Information:**

The online version contains supplementary material available at 10.1186/s12889-021-12083-y.

## Background

Early warning, situational awareness, and predictive information are all important for public health officials during infectious disease outbreaks. Traditional sources of infectious disease surveillance, such as sentinel surveillance, laboratory reporting, and case identification provide critical information for outbreak response, management, and decision-making. However, real-time and predictive outbreak information is often limited and can make it difficult for practitioners to respond effectively before an outbreak has reached its peak [1, 2]. In many cases, data collected through traditional surveillance methods often lags days or weeks behind an unfolding epidemic due to delays in collecting, reporting and analyzing data. Moreover, surveillance data may be abundant and timely for some epidemics or regions of the world, and poor and time-lagged for others, making it difficult to respond effectively across hazards and geographies. Given these and other challenges with traditional disease surveillance, it may be helpful to explore complementary approaches that have the potential to augment disease reporting and provide more forward-looking or predictive outbreak information. If early detection, tracking, and prediction of the course of an outbreak can be improved, public health practitioners and policy makers would be better able to respond to an outbreak and mitigate its effects on public health [3].

Crowd forecasting offers one possible approach to augmenting traditional infectious disease surveillance data to provide information on likely outcomes, as well as on uncertainty. A number of analytic modeling and crowd-sourced forecasting methods have emerged that could be applied to infectious diseases. Prediction markets, in which forecasters can buy and sell contracts related to outcomes, were first used for educational purposes at the University of Iowa beginning in 1988 [4–7]. Originally used to forecast political outcomes, currency prices, movie box office returns, and book sales, electronic markets have also been tested in forecasting health-related events [8, 9]. These markets aimed to aggregate diverse opinions and expertise in public health, medicine, and related fields, which would provide information that could improve response to infectious disease emergencies. In a pilot study on influenza prediction, participating health care professionals forecasted accurate levels of influenza activity 2–4 weeks prior to traditional, official surveillance reports [8]. Prediction markets focused on infectious disease have also been used to predict dengue outcomes in the United States and internationally [10].

One practical limitation of prediction markets is that many potential participants lack a background in commodities trading and, as a result, have difficulty expressing their forecasts. An alternative method of crowdsourcing forecasts for infectious disease surveillance is the use of prediction polls that aggregate individual forecasts statistically using recency-based subsetting, differential weighting based on past performance, and recalibration [11]. This method allows forecasters to make predictions using a more intuitive format in which they express beliefs by providing probabilities for potential outcomes. Outcomes are eventually resolved using ground truth and forecasters are scored on both accuracy and timeliness. In large-scale head-to-head comparisons of geopolitical forecasts, such prediction polls have proven to be as accurate as prediction markets [11]. Prediction polls are conducted to generate forecasts about future outcomes of interest and differ from classic “opinion polling.” Using prediction polling methods, the population of forecasters is not designed or expected to be representative of any specific population.

To test the utility of crowd-sourced knowledge for disease surveillance, the authors fielded a bespoke online forecasting tool that allowed a diverse set of experts to predict infectious disease outcomes. This research evaluated the types of disease outcomes, questions, and situations that would result in accurate forecasts. The ultimate goal was to develop an evidence base for the use of crowd-sourced forecasting to confidently provide information to decision makers that can supplement traditional surveillance and modeling efforts and improve response to infectious disease emergencies. Here we describe the development process for an online prediction polling platform and forecasting community as well as findings about the reliability, accuracy, and timeliness of the aggregated crowd forecasts.

## Methods

### Recruiting participants

Recruitment primarily targeted public health experts, medical professionals, epidemiologists, modelers, risk assessment experts, vector control officials, microbiologists, individuals with on-the-ground understanding of conditions surrounding disease outbreaks, public health graduate students, and others who were interested in infectious disease outbreaks. However, forecasting was open to any interested participant. The research team coordinated with ProMED-mail, an infectious disease reporting newsletter that reaches over 80,000 subscribers in at least 185 countries, as well as other infectious disease newsletters, professional networks, and public health groups [12]. Skilled prediction traders recruited and vetted by Hypermind over several years of participation in its geopolitical and business prediction market were also invited to join the project on a voluntary basis [13, 14]. Thirty one percent (31%) of the participants were recruited during the initial recruitment effort in January 2019. Another 51% of participants joined during a second recruitment drive in July 2019. Additional participants were allowed to join at any time over the 15-month course of the project. Although differences in starting date may have limited comparison between participants, allowing additional participants to join expanded opportunities to attract active participants and garner the most accurate forecasts – one of the primary goals of this project. Prizes were awarded in three rounds for two six-month periods (January–June, 2019; July–December, 2019) and one three-month period (January–March, 2020). The awards were based on forecasting performance (see “scoring participants” below). For the first and second rounds, the first-place prize was $599 with descending amounts awarded to a 5th place prize of $100. A performance-based raffle was used to award 20 additional participants $50 each. For the shorter third round, a similar prize structure was used with lower amounts starting at $500 and only including 12 raffle winners, since the competition did not run for as long.

### Developing the platform

**Fig. 1:**
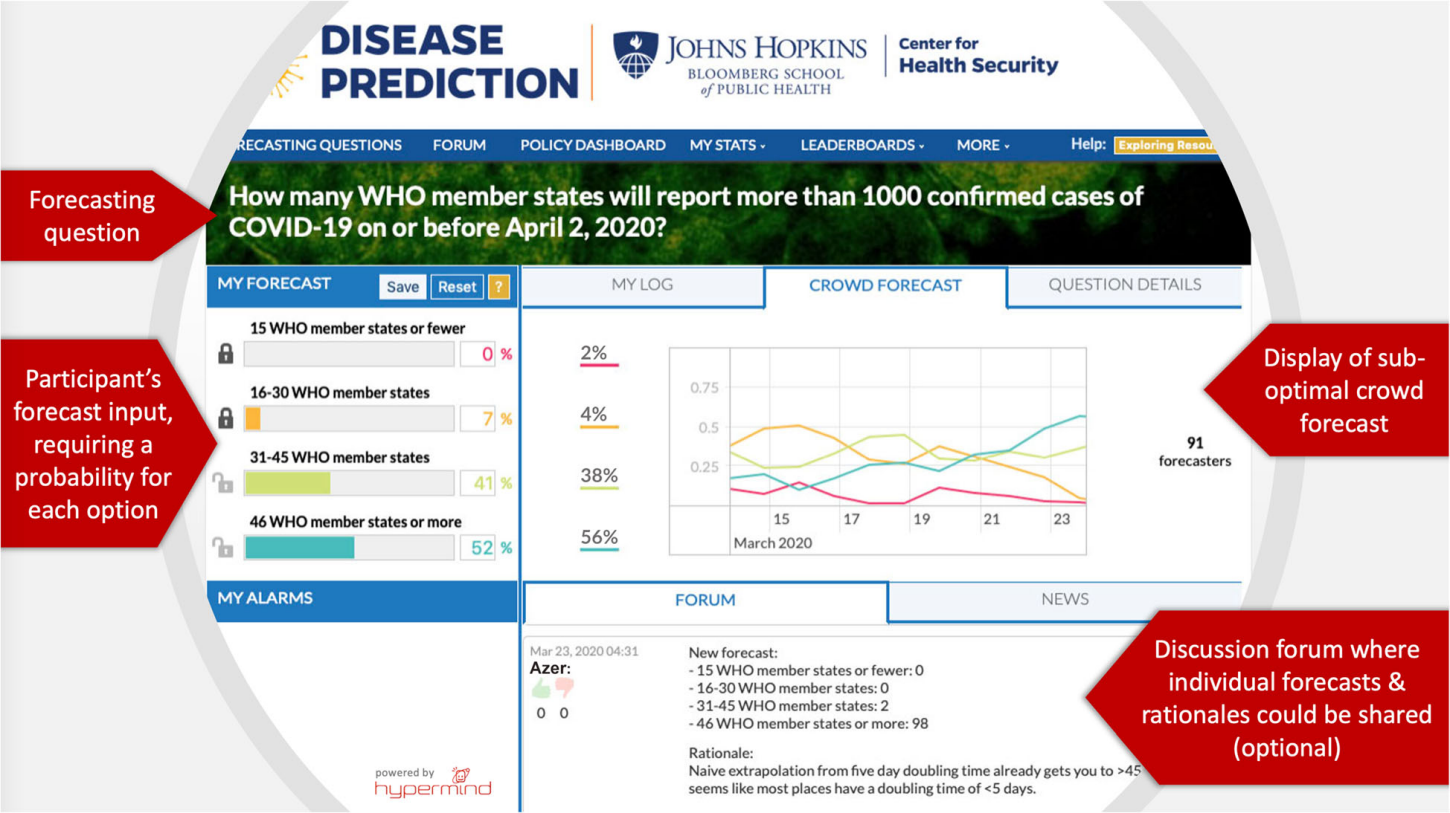
Main features of the forecasting user interface. Participants were asked to provide, for each question, a probability for each possible answer. The forecasting widget forced all probabilities to add up to 100%. After they made their first forecast in a question, participants were shown a current (but sub-optimal) aggregation of the collective (crowd) forecast for comparison, and could update their own at will. If they chose to, participants could also share their forecasts and rationales in the discussion forum

The research team explored a number of potential approaches to an online disease prediction platform and chose Hypermind (previously known as Lumenogic), a company with extensive experience in crowdsourcing techniques, to assist in this process [15]. After evaluating both prediction markets and prediction polls, the research team considered prediction polling through Hypermind’s *Prescience* platform to be the most easily accessible to those without experience in commodities trading, which was considered an important factor in attracting and retaining participants. Hypermind’s *Prescience* platform was developed though experience with several Intelligence Advanced Research Projects Activity (IARPA) research programs on crowd-based forecasting for the US intelligence community [ 16–18]. As Fig. 1 illustrates, the platform allowed participants to forecast easily and quickly by assigning probabilities to possible outcomes. They could update their forecasts as needed, share reasoning for their forecasts, engage in conversations with other forecasters, access open source information about disease topics, and compete for performance-based prizes. Importantly, forecasters were made aware of a current aggregated forecast of the crowd for each question, as well as its evolution over time, but only after having made their first forecast in the relevant question. The platform was also lightly customized for the particular needs of this project, including a bespoke dashboard aimed at policy makers.

### Developing forecasting questions

**Fig. 2:**
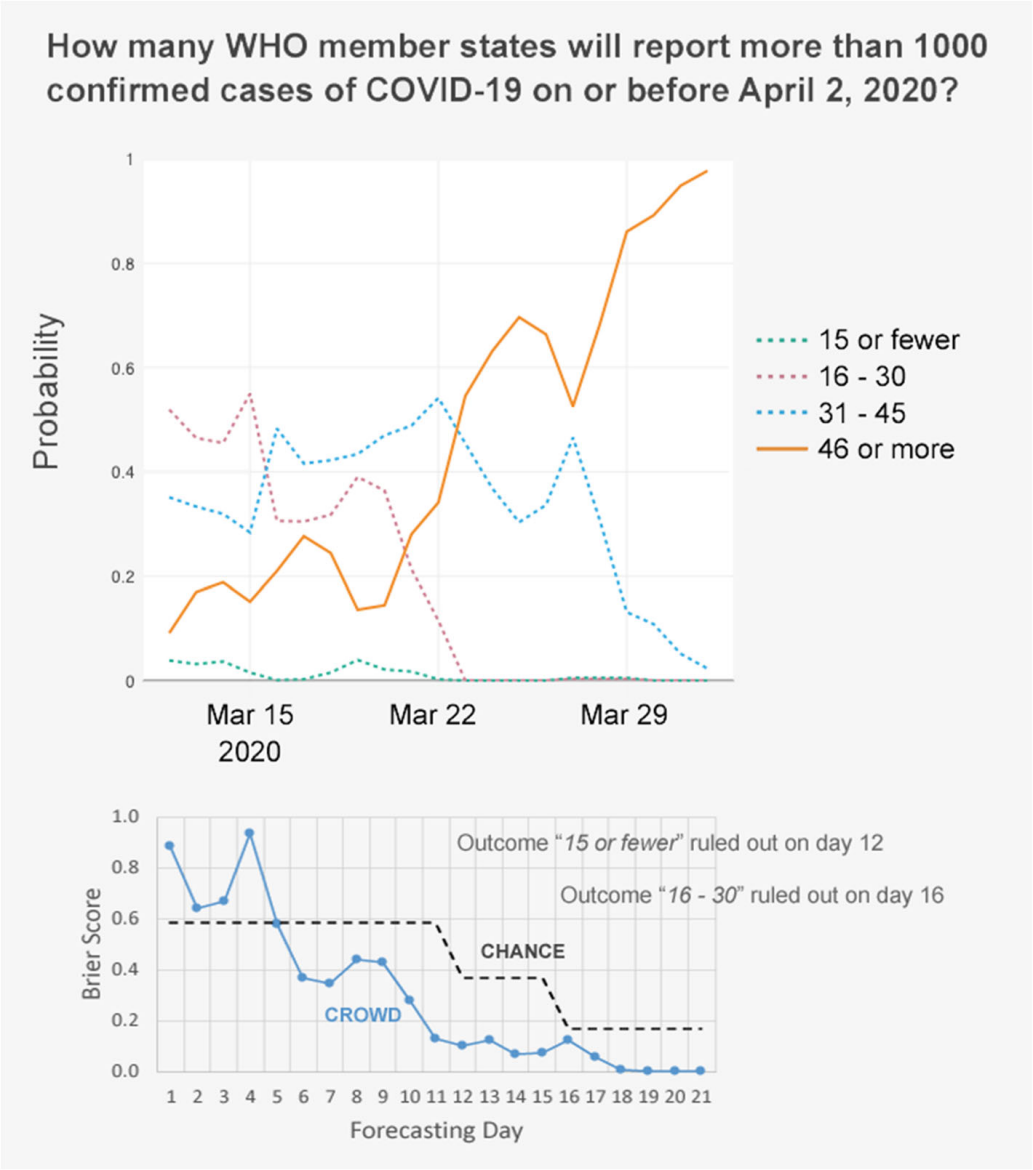
Example of an individual forecasting problem (IFP) of type “range”**.** This IFP was open to forecasting for 21 days. It featured 4 outcomes at the start, but one was ruled out by ground reports on day 12 and another on day 16. The top chart shows how the crowd-forecasted probabilities for each outcome evolved over time, with the solid line indicating the correct outcome (based on a ground truth of 48 WHO member states). The bottom chart shows the daily Brier scores (forecasting errors) of the crowd’s forecast as compared to the “chance” forecast which assigned equal probabilities to all outcomes not yet ruled out

The research team developed an initial set of questions for the platform and added new questions, or “Individual Forecasting Problems” (IFPs), at a rate of approximately 1 per week. [See Supplementary material] New IFPs were generally added on Mondays, in conjunction with a weekly newsletter, to encourage continued interest and participation in the project. IFPs were focused on a range of infectious disease outcomes, including intensity of disease (e.g. number of US states with high influenza-like illness activity), spread of disease to different locations, and case counts. When developing IFPs, care was given to ensure that the wording had only one possible interpretation, that forecasters would be able to select a discrete outcome from a complete set of mutually-exclusive answers weeks or months ahead of its occurrence, and that the IFP could be fairly resolved by a pre-identified and authoritative source that provided timely information (i.e. if an IFP asked for a case count by a certain date, the resolution source needed to provide a reliable report on that date). The platform allowed the posting of two types of IFPs: “discrete” IFPs featured two or more discrete answers (e.g., yes/no, or Beni/Butembo/Katwa/Mandima), while “range” IFPs featured three or more interval answers arranged on a continuum (e.g., 20 or fewer cases/21–100/101–300/more than 300 cases). Figure 2 shows an example of a “range” IFP.

### Scoring participants

The forecasting performance of each participant was measured relative to other participants’ for both timeliness and accuracy. Probability forecasts were scored using the Brier score [19] for discrete IFPs and its distance-sensitive ordered-categorical version [20] for range IFPs. Every day, the platform recorded each participant’s latest forecast for each IFP. If a participant had not made a forecast that day, his/her forecast from the previous day was carried over. When an IFP resolved according to ground truth, the score of each daily forecast was computed and compared to the median score of all other participants for that IFP on that day. Forecasts that were more accurate than the median led to point gains, while forecasts that were less accurate than the median caused forecasters to lose points. A participant whose score matched the median on a particular day scored 0 points on that day. On days before one had started forecasting an IFP, his/her daily score was imputed to be the median score obtained by all active forecasters, for better or worse. So as soon as one thought she could make a forecast that was better than most, she had incentives to do so.

### Aggregating the crowd forecast

Individual forecasts for each question were aggregated using an algorithm developed and tested through experience with several IARPA research programs in geopolitical crowd forecasting [11]. Individual forecasts were first weighted, with greater weight given to forecasters who update their predictions frequently and who have a better past record of accuracy. The pool of individual forecasts was then reduced so that only the 30% most recent forecasts were retained for aggregation, while others were discarded. The weighted forecasts were then averaged. Finally, an extremization step was used to sharpen the resulting forecast and compensate for collective under-confidence [21]. As previously noted, individual forecasters had access to a crowd forecast while making their own, but that publicly-displayed crowd forecast reflected a degraded version of the full algorithm just described. It was the simple average of the 30% most recent forecasts in that IFP, not taking into account individual weights nor extremization. We wanted the forecasters to position themselves relative to the crowd’s opinion without giving them the benefit of the fully-optimized crowd wisdom.

### Evaluating the crowd forecast

The crowd forecast’s absolute accuracy for each IFP was computed by averaging its daily Brier scores over the lifetime of the IFP. The overall accuracy of the aggregated forecasts was also computed as the average of its scores across all IFPs. But forecasting accuracy is only meaningful when compared to benchmarks, such as the “chance” forecast that would result from assigning equal probabilities to all possible outcomes in an IFP, or the accuracy of the individual forecasters themselves. The accuracy and timeliness of the crowd forecast were further evaluated in four increasingly severe ways: 1) the percentage of the lifetime of an IFP that the crowd forecast was more accurate than chance, 2) the point in the lifetime of an IFP at which the crowd forecast became irreversibly better than chance (the earlier the better), 3) the percentage of the lifetime of an IFP that the correct outcome was the crowd’s favorite, and 4) the point in the lifetime of an IFP at which the correct outcome became irreversibly the crowd’s favorite. For example, in the IFP described in Fig. 1, the crowd’s forecast was better than the chance forecast for 16 days out of 21, or 76% of the lifetime of that IFP. It became irreversibly better than the chance forecast on day 5, or 24% into the lifetime of that IFP. The crowd favorited the correct outcome for 10 out 21 days, or 48% of the lifetime of that IFP. The correct outcome became irreversibly the crowd’s favorite on day 12, or 57% into the lifetime of this IFP.

## Results

### Individual forecasting problems

Over the course of 15 months (January 2019 – March 2020), 61 IFPs were provided to forecasters and eventually settled: 15 (25%) were of the discrete kind and 46 (75%) were of the range kind. They featured 2 to 6 possible outcomes, with an average of 3.56 outcomes per IFP. In total, 217 possible outcomes were forecastable for the 61 settled IFPs. The average IFP lifetime was 47 days (median 31.5 days) from opening to settlement, but that metric varied widely across IFPs, from 7 to 289 days. At most, 10 IFPs were concurrently active on the platform at any time.

### Forecasters

Over the course of the project, 562 participants forecasted on at least one IFP. On average, IFPs had participation from 92 participants (range: 35–252 forecasters) and participants forecasted on 10 IFPs. In total 10,750 forecasts were collected over the 61 settled IFPs (where each forecast features a probability estimate for each of the outcomes listed in the relevant IFP). While a small majority of participants, 54%, were public-health professionals, 15% had professional backgrounds in other health-related fields, and the remaining 31% did not report any health-related professional background. Of the 132 skilled forecasters vetted by Hypermind who participated, only 5 were public-health professionals, and only 6 more indicated some other health-related professional background. Both kinds of “expertise”, in relevant domain knowledge or in general-purpose forecasting skill, seem to have powered quality forecasting of disease outbreaks [22]. For instance, among the top 10 best forecasters in the contest’s final leaderboard, 4 were public-health professionals, 3 had some other health-related professional background, and 3 were Hypermind skilled forecasters who did not report any health-related professional background. From another angle, five were vetted Hypermind forecasters, while the other 5 were not. Furthermore, the 1st place forecaster was one of the very few public-health professional who was also a Hypermind skilled forecaster.

### Crowd forecast reliability (calibration)

**Fig. 3:**
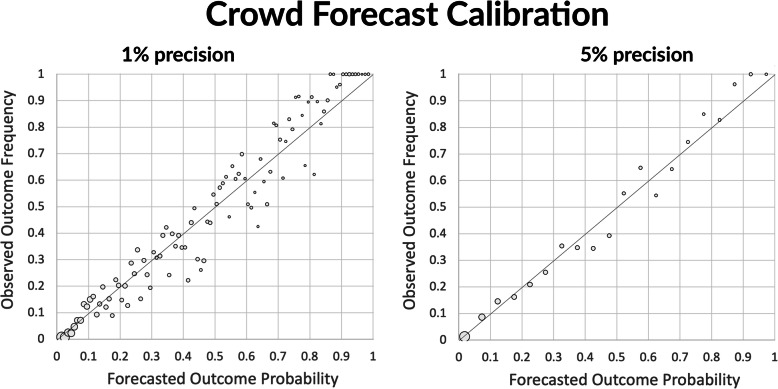
Crowd forecast reliability. The charts plot the proportion of forecasts with probability *p* that correspond to outcomes that occurred. The left plot shows the data at the 0.01 probability level of precision. In the right plot, all the outcomes forecasted at probabilities [.01, .05] are grouped together into a single data point, then all the outcomes forecasted with probabilities within [.06, .10], etc. This grouping by successive chunks of 5% helps reduce the noise in the data. The closer the data points align with the diagonal, the more reliable the forecast probabilities are. The surface area of each dot indicates relative number of forecasts made at that level of probability. A greater proportion of forecasts were made at lower probabilities because the majority of IFPs featured 3 or more outcomes, only one of which could eventually occur

We evaluated the crowd’s forecast calibration, or the consistency between the crowd’s forecasted probabilities and the observed outcome occurrences, expecting approximately 20% of all forecasts made with a probability of 0.20 to correspond to outcomes that occurred; 30% of those made with a probability of 0.30 to correspond to outcomes that occurred, etc. If the forecasts were perfectly calibrated, the data points in Fig. 3 would perfectly align on the diagonal. To formally compute calibration, we used Murphy’s reliability score, a well-established standard in weather forecasting [23] and more recently in geopolitical forecasting. However, given that some IFPs were forecasted up to 40 times longer than others and attracted up to 20 times more forecasts, we first weighted each IFP’s forecasts by the inverse of the number of forecasts in that IFP. The resulting crowd forecast reliability measures were .0043 and .0015 respectively at the 1 and 5% levels of precision.

### Crowd forecast accuracy and timeliness

When experimenting with crowd wisdom a classic benchmark is to compare the accuracy of the crowd against that of its individual members. A fair comparison required that we restrict the IFP sample to the 54 that were added to the platform after the Hypermind forecasters were invited to join (which was 3 weeks after the start of the project). On this sample, the mean and median forecasters achieved Brier scores no better than chance (.466 and .465 vs .465), and only 6 individuals achieved lower Brier scores than the unweighted average of everyone’s forecasts (.331). Of those, only 3 bested the unweighted average of the 30% most recent forecasts that was displayed in the platform’s user interface (.276), and none outperformed the optimized crowd forecast computed by the full aggregation algorithm (.245).

Over all 61 IFPs, the optimized crowd aggregation (henceforth simply referred to as the crowd forecast) was 48% more accurate than chance overall (Cohen’s d = 1.05). The average of its mean daily Brier score across all IFPs was 0.238 as compared to a score of 0.460 that would result from chance. Of 61 IFPs, there were only 6 in which the crowd forecast was less accurate than chance (with no discernible common link among them). Because the crowd Brier scores were very skewed (1.37), a Wilcoxon Signed-Ranks Test was run and the output indicated that the crowd aggregation advantage over chance forecasting was highly significant (Z = 1774; *p* < .001).

**Fig. 4:**
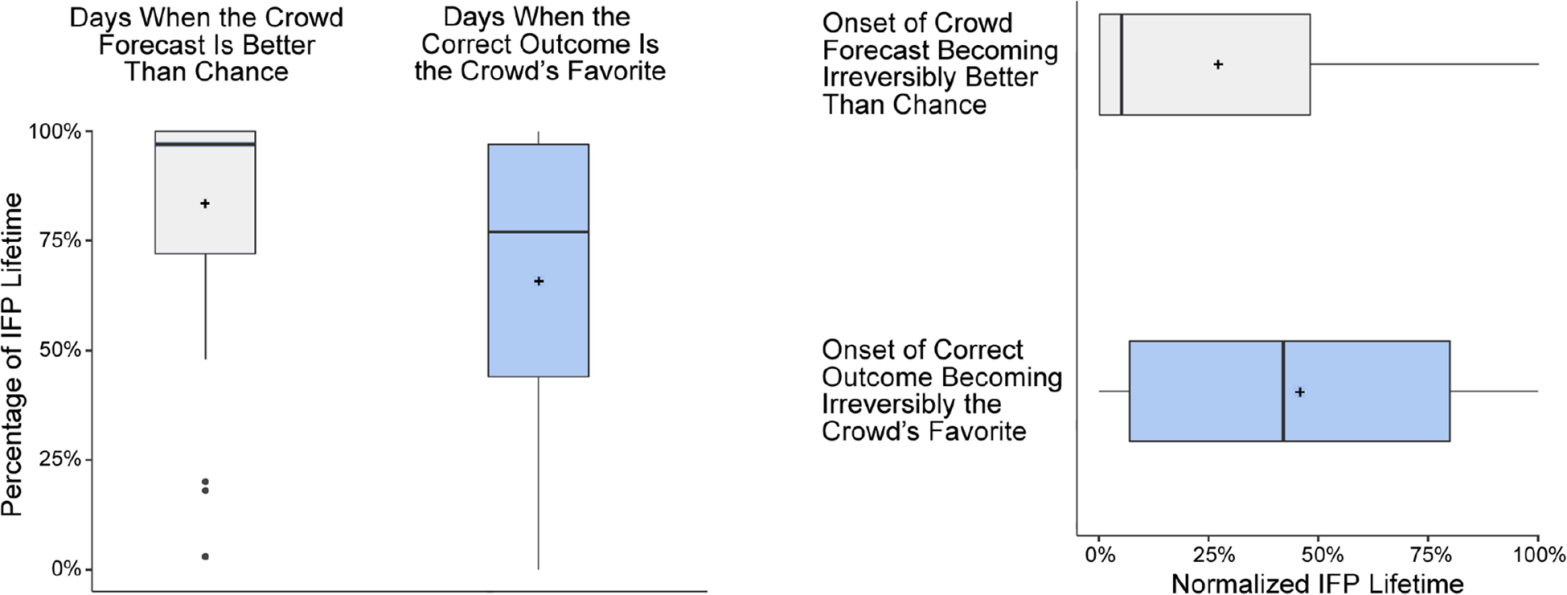
Four measures of the accuracy and timeliness of the crowd’s forecasts. The left chart plots *the percentage of days* in the lifetime of each IFP that the crowd’s forecast met one of two accuracy criteria. The right chart plots *how early* in the lifetime of each IFP the crowd’s forecast met one of two accuracy criteria. The box plots represent 61 IFPs, with mean values are indicated by “+” marks. See text for a detailed description

Figure 4 shows how well and how early the crowd’s forecast met two relevant accuracy criteria: the first criterion is being more accurate than a chance forecast (equal probabilities across all possible outcomes). The second criterion, which is more severe, is assigning the highest probability to the correct outcome. The left plot shows that for most of the IFPs the crowd’s accuracy was better than chance for almost the entire lifetime of the IFP (median: 97% of the lifetime of an IFP; mean: 83%). Furthermore, the correct outcome was the crowd’s favorite for most of the lifetime of most IFPs (median: 77% of the lifetime of an IFP; mean: 66%). In terms of timeliness, the right plot in Fig. 4 shows that the crowds’ accuracy generally became *irreversibly* better than chance (i.e. from that point in time until ground-truth was observed) very early in the lifetime of most IFPs (median 5% into the lifetime of the IFP; mean: 27%). Furthermore, for most IFPs, the correct outcome became the definitive favorite of the crowd in the first half of the IFP’s lifetime (median: 42% into the lifetime the IFP; mean: 46%).

### Question formation

The types of IFPs proposed impacted forecaster accuracy. Selecting the correct interval in a range IFP (75% of the questions) is intrinsically more difficult than selecting the correct choice in a discrete IFP. In several instances we observed that the correct outcome alternated between the 1st and 2nd highest assigned probabilities, with the nearest competing outcome being closely related (e.g. the next lowest or next highest level of case counts offered as a potential answer). Six IFPs, not included in the analytical sample of 61 settled IFPs, were voided due to uncertainty in interpretation or resolution.

## Discussion

Over the 61 settled IFPs, crowd forecasts showed high levels of reliability and accuracy. However, instances in which the crowd did not produce definitive outcomes in a timely manner were useful as well. In these cases, the inability of the crowd to decide on a single outcome highlighted substantial levels of uncertainty related to future directions of the outbreak or disease in question. Providing information on levels of certainty is also a valuable component in decision making during infectious disease events.

The most difficult component of fielding this infectious disease prediction platform was the development of forecasting questions. Questions had to be carefully designed to be straightforward and simple enough to have a limited number of possible outcomes and, at the same time, complex enough to provide useful information to policymakers and public-health practitioners. Furthermore, outcomes had to be published by reputable sources for the time frame in question. This was a difficult balance to strike, and more refinement is required to develop lines of inquiry that are simple to interpret, readily resolved, and easily used for decision-making during an outbreak.

Frequent and accurate public health surveillance data is needed to enable research team members to develop relevant questions and for forecasters to make accurate predictions. The project team observed that forecasters seemed to perform better on questions covering topics with reliable and frequently updated official surveillance data, high levels of media coverage, and details about cases. Formulating appropriate answer ranges, identifying the correct time period for questions to resolve, determining accurate question resolutions and understanding overall disease dynamics requires an accurate starting point based on accurate and frequent disease reporting. Final surveillance information is only needed for scoring purposes, but without this information, it would be difficult to provide feedback to forecasters on how accurate their forecasts were, limiting incentives for thoughtful forecasting.

Through the question development process, the research team identified only a few countries and regions with publicly available, timely, and reliable disease reporting. It was relatively rare for countries and regions to issue reports on a regular and predictable schedule. Furthermore, the quality of reporting within a region or country can vary widely depending on the disease or outbreak context (e.g., animal vs human disease). Poor reporting greatly limited opportunities to ask forecasters to predict on disease outbreaks, especially those that were newly emerging. Paradoxically, those situations with the least robust disease surveillance are those that could most benefit from supplementary information from crowd forecasting.

Additional research is needed to ensure crowd forecasting information can be translated into meaningful actions by public health and other response officials. For instance, several IFPs focused on the speed and geographic spread of the virus that causes COVID-19, showing the potential for rapid escalation of a global pandemic as it emerged. This type of information must be meaningfully merged into existing data streams and systems to enable decision making.

The forecasting platform was established in early 2019 as a proof-of-concept project to understand more about crowd forecasting using prediction polling for infectious disease outcomes. As a result, it was operating with a large number of infectious disease and forecasting experts during the emergence of COVID-19. The final round of forecasting included questions focused heavily on the emerging pandemic. The crowd accurately predicted explosive growth and spread of the disease but forecasts in some instances also provided indications of uncertainty, likely due to poor disease reporting, testing, and surveillance early in the outbreak. Establishing standing crowd forecasting efforts could aid in rapidly producing predictions for emerging outbreaks. Obviously, during emerging outbreaks, those with professional public-health responsibilities may be less able to participate in and conduct such efforts. However, the remaining crowd of skilled forecasters and professionals in other health-related fields can continue to provide timely well-informed forecasts. That is especially true when large numbers of public-health professionals are suddenly focused on documenting and publishing timely information about the outbreak, as was the case in the early stages of the Covid-19 pandemic.

### Limitations

This project was subject to a number of limitations. The project team worked hard to develop IFPs on topics without an obvious answer, but difficulty varied. This process also required forecasting by the project team to identify appropriate questions and ranges. In retrospect, IFP answer options in several cases should have included higher ranges. Ideally, the forecasting task should be left entirely to the forecasters, not to those asking questions. This issue can largely be addressed by further technical development of the forecasting platform, which we have begun experimenting with. The use of monetary rewards may have influenced participant behavior. However, the level of the award was unlikely to be large enough to lead to meaningful shifts in forecasts.

## Conclusion

Over the 61 settled IFPs, crowd forecasts showed high levels of reliability, accuracy and timeliness. Consistent with the “wisdom of crowds” phenomenon, crowd forecasts aggregated using best-practice algorithms proved well-calibrated and outperformed all individual forecasters, a majority of which had professional expertise in public-health. Crowd forecasting efforts in public health may be a useful addition to traditional disease forecasting, modeling, and other data sources in decision making for public health events. Such crowd-sourced forecasting can help to predict disease trends and allow public health and policymakers time to prepare and respond to an unfolding outbreak. These efforts will never replace traditional surveillance methods, since surveillance data is the gold standard and is also needed to verify prediction platform outcomes, but they can supplement traditional methods. By providing rapid synthesis of the knowledge and expectations of experts and informed amateurs, crowd-sourced forecasting can help inform decision-making surrounding implementation of disease mitigation strategies and predict where disease may cause problems in the near future. While promising in concept and in pilot testing, prediction polling for infectious diseases should be tested further with a particular focus on determining optimal participant make-up, understanding the best incentive structure, optimizing participant experience and asking questions that are most timely and relevant to policymakers.

## Supplementary Information

**Additional file 1.**

## Data Availability

The data generated during the study are not publicly available. They are currently being reviewed and evaluated for additional results in preparation but not yet published. Data may be made available upon reasonable request to the corresponding author.

## Abbreviations

IARPA: Intelligence Advanced Research Projects Activity
IFP: Individual Forecasting Problems
ProMED: Program for Monitoring Emerging Diseases
WHO: World Health Organization
